# How equitable are the distributions of the physical activity and accessibility benefits of bicycle infrastructure?

**DOI:** 10.1186/s12939-021-01543-x

**Published:** 2021-09-15

**Authors:** Christopher Standen, Melanie Crane, Stephen Greaves, Andrew T. Collins, Chris Rissel

**Affiliations:** 1 Centre for Primary Health Care and Equity, School of Population Health, University of New South Wales, Kensington, NSW 2052 Australia; 2 Health Equity Research and Development Unit, Sydney Local Health District, Missenden Road, PO Box M30, Camperdown, NSW 2050 Australia; 3 Sydney School of Public Health, Charles Perkins Centre, The University of Sydney, Camperdown, NSW 2006 Australia; 4 Institute of Transport and Logistics Studies, The University of Sydney Business School, The University of Sydney, Darlington, NSW 2006 Australia

**Keywords:** Health equity, Physical activity, Accessibility, Bicycle

## Abstract

**Background:**

Cycling for transport provides many health and social benefits – including physical activity and independent access to jobs, education, social opportunities, health care and other services (accessibility). However, some population groups have less opportunity to reach everyday destinations, and public transport stops, by bicycle – owing in part to their greater aversion to riding amongst motor vehicle traffic. Health equity can therefore be improved by providing separated cycleway networks that give more people the opportunity to access places by bicycle using traffic-free routes. The aim of this study was to assess the health equity benefits of two bicycle infrastructure development scenarios – a single cycleway, and a complete network of cycleways – by examining the distributions of physical activity and accessibility benefits across gender, age and income groups.

**Methods:**

Travel survey data collected from residents in Sydney (Australia) were used to train a predictive transport mode choice model, which was then used to forecast the impact of the two intervention scenarios on transport mode choice, physical activity and accessibility. The latter was measured using a utility-based measure derived from the mode choice model. The distributions of the forecast physical activity and accessibility benefits were then calculated across gender, age and income groups.

**Results:**

The modelled physical activity and accessibility measures improve in both intervention scenarios. However, in the single cycleway scenario, the benefits are greatest for the male, high-income and older age groups. In the complete network scenario, the benefits are more equally distributed. Forecast increases in cycling time are largely offset by decreases in walking time – though the latter is typically low-intensity physical activity, which confers a lesser health benefit than moderate-intensity cycling.

**Conclusions:**

Separated cycleway infrastructure can be used to improve health equity by providing greater opportunities for transport cycling in population groups more averse to riding amongst motor vehicle traffic. Disparities in the opportunity to access services and economic/social activities by bicycle – and incorporate more physical activity into everyday travel – could be addressed with connected, traffic-free cycleway networks that cater to people of all genders, ages and incomes.

## Background

Transport is one of the main social determinants of health [1]. The way transport systems are designed, and the resources allocated to them, have the potential to disproportionately benefit or negatively impact certain population groups or neighbourhoods [2]. For example, building a transport system that prioritises private motor vehicles makes getting around more convenient – though less healthy and enjoyable [3] – for those people who can afford to own, and are able to drive, motor vehicles. Meanwhile the external effects of motor vehicle traffic, such as air and noise pollution, affect the health of disadvantaged groups more [4].

Giving more people the opportunity to ride a bicycle for everyday transport – through providing connected networks of quiet streets and paths protected from motor vehicle traffic – has well-documented public health, sustainability and economic benefits [5–7]. However, the health equity impacts of bicycle infrastructure projects or plans are rarely assessed. While there is a considerable literature on the equity of road pricing schemes and public transport, there is a smaller body of knowledge on the equity impacts of bicycle policies. The literature here includes studies on the use of bicycle share schemes by different population groups [8], but has largely failed to assess health aspects, including health equity [9].

The health benefits and costs measured in assessments of active transport interventions (e.g., new walking and cycling infrastructure) typically include changes in physical activity, road trauma and air pollution exposure [5, 10, 11]. One example of a health equity-focused assessment of active transport infrastructure is that by Wu et al. [12], who developed a model to forecast changes in disability-adjusted life years attributable to changes in physical activity and road trauma, across race/ethnicity and income groups.

In Australia, cycling for transport is often viewed as the preserve of male, inner-city white-collar workers [13]. This view is supported by Census data, which show that bicycle commuters are most likely to have an above-average income, be male, and be aged 20 to 49 years [14–16]. However, it is older adults and females who are more likely to be inactive or only moderately active (Australian Bureau of Statistics, 2019), and therefore have most to gain – physical activity-wise – from having more opportunities to cycle for everyday transport. Furthermore, the development of transport-oriented bicycle paths and networks (as opposed to recreational paths and trails) has been concentrated in gentrified, inner-city areas. Yet, it is outer-suburban residents who are less likely to be achieving sufficient physical activity to produce a health benefit [17].

Given the physical and mental health consequences of social/economic isolation and loneliness [18], and poor access to services (including health care) [19], another important benefit of bicycle infrastructure is improved accessibility, i.e., more opportunities to access jobs, education, social opportunities, services, healthy food options, etc. There are several established ways of measuring accessibility [20]. For example, contour measures simply count the number of opportunities that can be reached within a given travel time or distance of an origin. Utility-based measures are founded in welfare economics and attempt to place a monetary value on the range of destination and mobility choices available to an individual. Kent and Karner [21] used a contour measure to assess how accessibility to supermarkets, libraries and businesses would improve with a range of proposed bicycle infrastructure projects – and how these accessibility improvements would be distributed according to poverty status, race, and motor vehicle ownership status. We are aware of no equity-focused health assessment of an active transport intervention that has measured changes in accessibility using a utility-based measure.

## Methods

### Aim

**Fig. 1:**
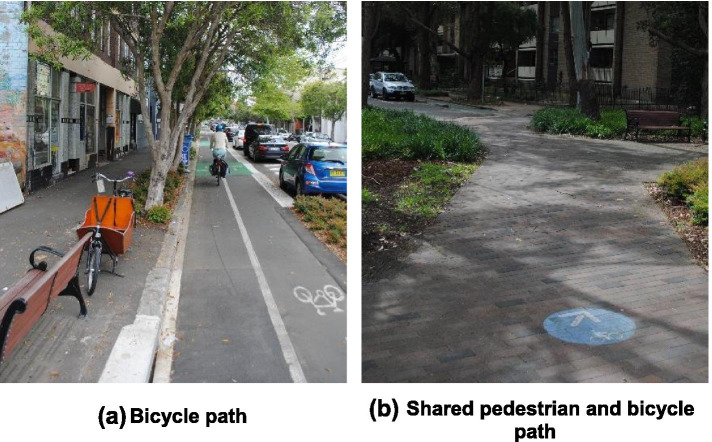
Examples of cycleway infrastructure

In this study, we assess the health equity impacts of two planned bicycle infrastructure interventions in Sydney (Australia) – a single new cycleway, and a complete, connected network of cycleways. We define ‘cycleway’ as infrastructure comprising traffic-protected bicycle paths (also known as bicycle tracks) and/or shared pedestrian and bicycle paths (see Fig. 1).

### Setting

The study area was the City of Sydney local government area (LGA), which is situated in the eastern part of the Greater Sydney metropolitan area in the Australian state of New South Wales (NSW). It comprises Sydney’s main central business district (CBD) and the surrounding inner-city suburbs. It has a diverse population and has experienced significant gentrification in recent years, though pockets of socio-economic disadvantage remain.

Greater Sydney is car-oriented and not conducive to everyday transport cycling due to a lack of traffic-protected cycling facilities and a high default residential speed limit (50 km/h) [22]. State laws also mandate the use of bicycle helmets, which can present a barrier to participation in transport cycling [23]. These laws are zealously enforced, with young Aboriginal and Torres Strait Islander peoples – who are less likely to have the means to pay the A$344 fine – targeted in particular [24, 25]. Despite these laws, cycling injury risk is relatively high for a high-income country [26, 27].

Some roads and streets have paint-marked bicycle lanes, but these are often situated in the hazardous ‘door zone’ between parked vehicles and general traffic lanes. At the time of data collection, the few existing cycleways were disconnected and lacked continuity, with very poor level of service (i.e., long waiting times) at signal-controlled intersections. The City of Sydney LGA is relatively hilly, which may partly explain the boom in e-bike sales in recent years [28]. The climate is temperate with hot, humid summers and mild winters, and an average of 144 rainy days and 1,211 mm of rain per year [29].

On the day of the 2016 Census, 4% of males and 1.8% of females in the City of Sydney LGA commuted to work using a bicycle as their main transport mode. The respective values for Greater Sydney were 1.1 and 0.3% [30].

As part of a policy to give more people the opportunity to use a bicycle for everyday transport, City of Sydney Council has a Cycle Strategy and Action Plan [31]. The centrepiece of this strategy is a planned 200 km bicycle network, including 55 km of cycleways.

### Approach

Lee et al. [32] developed a theoretical framework for assessing the broad equity impacts of active transport policies and plans, which is summarised in Table 1. The first step within this framework is to choose a model of distributive justice, i.e., whether resources should be targeted to address inequality, inequity or need [33]. The second step is to choose the equity lens(es), e.g., whether to assess the distribution of benefits and costs between different population groups or between geographic areas. The final step is to select the individual benefits and/or costs to measure.

**Table 1:** Theoretical framework for assessing equity in active transport planning – adapted from Lee et al. [32]

**Models of distributive justice**	***Equality rule***	• Benefits and costs of active transport should be the same for everyone.
	***Equity rule***	• Benefits and costs of active transport should be distributed proportionally, e.g., provide infrastructure where demand is highest.
	***Needs rule***	• The greatest benefit should be provided to the most disadvantaged population groups or geographical areas.
**Approaches to identifying inequities in measured benefits or costs of active transport.**	***Social***	• Assesses how active transport benefits or costs are distributed between different population groups. • Focus is typically on disadvantaged population groups, e.g., low income, indigenous, females. • Disadvantaged population groups sometimes have the most to gain from active transport policies, due to lower levels of physical activity, motor vehicle ownership and access to public transport.
	***Spatial***	• Assesses how active transport benefits or costs are distributed between different geographical areas (e.g., neighbourhoods).
	***Modal***	• Assesses whether users of a given mode of transport are better/worse off than others, or disproportionately affected by a transport policy or project, e.g., pedestrians having longer average waiting times at signal-controlled intersections.
	***Procedural***	• Assesses the fairness of decision making, e.g., whether disadvantaged groups/areas/modes are considered in, or disproportionately affected by, strategies, plans, designs, etc.
**Measures of the benefits and costs of active transport.**	***Benefits***	• Availability or accessibility of active transport infrastructure. • Accessibility to employment, education, public transport stops, supermarkets and other activity destinations. • Active transport infrastructure quality, e.g., kerb ramps and pavement quality. • Physical activity associated with active transport.
	***Costs***	• Exposure to air pollution. • Risk of being killed/injured by a motor vehicle driver.

**Fig. 2:**
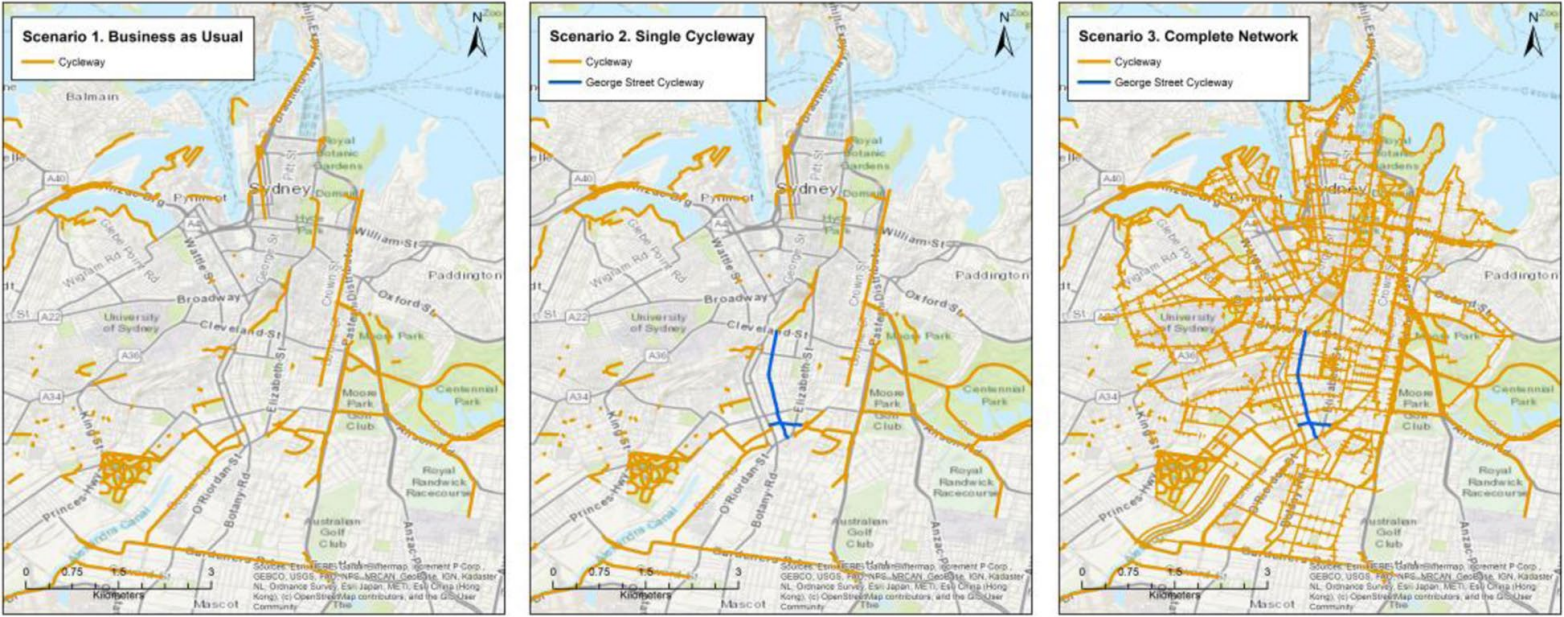
Scenario maps

Within this framework, we adopt the ‘needs rule’ with a ‘social’ equity lens – assessing how the health benefits of the two infrastructure intervention scenarios (relative to a *Business as Usual* scenario) are distributed across gender, age and income groups. The three scenarios are described in Table 2 and mapped in Fig. 2. The health benefits we measure are changes in physical activity and accessibility, which we forecast using a predictive transport mode choice model.

**Table 2:** Scenario descriptions

**Scenario**	**Description**
*Business as Usual*	The bicycle network as it existed in 2013.
*Single Cycleway*	The 2013 network, plus a single 2.4 km cycleway along George Street, connecting the Green Square urban renewal area in Sydney’s Inner South with the CBD, and passing through suburbs with a large amount of public housing and Aboriginal Housing Office housing (Waterloo and Redfern).
*Complete Network*	The 2013 network, plus completion of the bicycle network proposed in the Cycle Strategy and Action Plan [31].

### Sample

Data collection is described in detail elsewhere [34] but, for the benefit of the reader, a brief synopsis follows. Participants were recruited between September and November 2013. Eligibility was based on residential location (City of Sydney LGA only), age (18–55 years only) and self-reported ability to ride a bicycle. Recruitment was via several methods, including consumer panels, random digit dialling, letterbox drops, social media and electronic mailing lists.

Following recruitment, participants were asked to complete an online questionnaire and seven-day travel diary. Participants were given the option to download a smartphone tracking app to record their travel and assist them in completing their travel diaries. Those completing both the questionnaire and the travel diary were given a financial reward of $A65.

### Variables

In the questionnaire, participants were asked about their age, gender, household income, and education level. For the gender question, only two response options were available (female and male).

Physical activity in the previous week was measured using the Active Australia Survey [35]. Participants were also asked what type of bicycle rider they most identified as [36]. Response options were:

- ‘A low-intensity recreational bike rider – you like the fresh air and exercise, and cycle at an enjoyable pace’;
- ‘A high-intensity recreational bike rider – you like to ride hard and fast’;
- ‘A low-intensity transport bike rider – you are about just getting to places, and you travel at a more comfortable speed’; and
- ‘A high-intensity transport bike rider – you are a fast rider who likes to keep up a fast pace throughout your journey’.

For analysis and presentation purposes, this variable was dichotomised as ‘high intensity’ and ‘low intensity’.

The seven-day travel diary collected, for each activity of each day: activity type (e.g., ‘commute to work/study’ or ‘shopping’), mode of transport, access and egress modes of transport (for public transport trips), origin, destination, departure time and arrival time [37].

Daily rainfall data were obtained from the Bureau of Meteorology [38].

### Analysis

Analysis comprised three main steps: (1) using the collected data to train a predictive transport mode choice model; (2) using this model to forecast the impact of the two intervention scenarios on transport mode choice, physical activity and accessibility; and (3) assessing how the forecast physical activity and accessibility changes are distributed across gender, age and income groups. More detail on each step is provided below.

#### Predictive transport mode choice model

For each trip reported in the travel diary, the travel time/distance attributes (i.e., features or independent variables) of four transport mode alternatives (walk, bicycle, public transport and car) were imputed from the reported origin and destination, using ArcGIS Network Analyst software [39] and the Google Directions application programming interface [40].

Mode choice (the label/dependent variable) was coded as the reported transport mode for the trip – or, for multimodal trips, the mode with the highest priority in the hierarchy used by the NSW Bureau of Transport Statistics [41].

For each choice situation (i.e., trip) *t*, it was assumed that the observed utility ${V}_{njt}$ (i.e., the relative attractiveness) of transport mode alternative *j* to individual *n* is given by:

1 $$V_{njt} = \alpha_{j} + \beta^{\prime}_{n} x_{njt} ,\;t = 1,\; \ldots ,\;T_{i} ,$$

where: ${x}_{njt}$ is a vector of individual characteristics (e.g., age), trip attributes (e.g., cycleway distance), contextual factors (e.g., daily rainfall below/above 3 mm) and interaction terms (e.g., non-cycleway distance multiplied by high/low-intensity bicycle rider); $\beta^{\prime}$ is a vector of parameters to be estimated; and $\alpha_j$ are alternative-specific constants.

The mixed logit model was chosen because it can account for panel data, i.e., correlation between multiple choice situations (trips) for one individual. Separate models were estimated for commuting and non-commuting trips. The alternative-specific constant for the walk alternative was normalised to zero. For the random parameters, it was found that a triangular distribution – with spread constrained to be half the mean – gave the best behavioural interpretation. Models were estimated by simulated maximum likelihood using NLOGIT version 6 choice modelling software [42].

The modelled attributes of bicycle utility included distance, which was broken down into cycleway distance and non-cycleway distance. Thus, if the parameter estimate for non-cycleway distance is more negative than that for cycleway distance, then interventions that allow more of a trip to be undertaken on cycleways will increase the utility of cycling, and the probability ${P}_{nt}$ of bicycle being chosen for that trip:

2 $$P_{nt,j=bicycle}=\frac{exp\left(V_{nt,j=bicycle}\right)}{exp\left(V_{nt,j=walk}\right)+exp\left(V_{nt,j=bicycle}\right)+exp\left(V_{nt,j=public\;transport}\right)+exp\left(V_{nt,j=car}\right)}.$$

The bicycle utility function also included as trip attributes: dummy variables for daily rainfall greater than 3 mm (*Rain* > *3 mm (Bicycle)*), and whether the trip began or ended in the CBD (*CBD (Bicycle)*). The car utility function included as trip attributes: travel time, and a dummy variable indicating whether the trip began or ended in the CBD (*CBD (Car)*). Only travel time was retained as a trip attribute in the walk and public transport utility functions. Non-statistically significant variables were omitted from the final models.

#### Forecasting

For all three scenarios, it was assumed that each participant would make the same trips they reported in their seven-day travel diary, with the same origins and destinations, and that the attributes for the walk, public transport and car alternatives would not change. For the bicycle alternative, the imputed cycleway and non-cycleway distances could differ in each scenario, due to the addition of new cycleways to the network.

For each trip in each scenario, the expected values of walking time and cycling time were calculated using a simulation model developed using Microsoft Excel [43] – with the probability of a transport mode being chosen calculated using Eq. (2) and daily rainfall simulated at random based on the historical rainfall data. Cycling time was derived from cycling distance, assuming an average cycling speed of 15 km/h (the value used by the United Kingdom Department of Transport for adults with limited cycling experience [44]). Differences in annual walking time and annual cycling time between each intervention scenario and *Business as Usual* were calculated and multiplied by 52.1 to obtain annual forecasts.

Following Train [45], de Jong et al. [46] and Geurs and van Wee [20], differences in utility-based accessibility ${A}_{nt}$ between an intervention scenario (*s* = *2*) and *Business as Usual* (*s* = *1*) were calculated as:

3 $$\Delta E\left({A}_{nt}\right)=\left(1/{\mathrm{\alpha }}_{n}\right)\mathrm{ln}\left(\sum_{j}{e}^{{V}_{ntj}^{s = 2}}\right)-\left(\sum_{j}{e}^{{V}_{ntj}^{s = 1}}\right).$$

The marginal utility of income $${\alpha }_{n}$$ is, by definition, the negative of the parameter of any monetary variable in a mode choice model, e.g., public transport fare [45]. Because there was no monetary variable in our model, a variable with a well-established monetary valuation was chosen; namely, the value of travel time savings, which the NSW Government valued at an average of $A15.14/hour [47].

#### Equity analysis

The resulting outcome variables of annual walking time (hours), annual cycling time (hours) and utility-based accessibility changes (A$) were aggregated by participant and averaged for each gender, age and income group (as listed in Table 3). Forecast changes in physical activity for each population group were compared across the two intervention scenarios using slope graphs. Forecast increases in cycling time and utility-based accessibility per person per year per kilometre of new cycleway were compared across population groups for each intervention scenario using grouped bar charts. This analysis was performed using Microsoft Excel [43] and Tableau Desktop [48].

**Table 3:** Sample characteristics

	**n**	**Average self-reported minutes of physical activity per week**	**% that are sufficiently physically active**	**Average (self-reported) body mass index (BMI)**^**a**^	**Average number of reported trips (seven days)**
**Gender**					
Female	212 (79%)	557	95	20.9	18.7
Male	55 (21%)	533	82	26.3	17.7
**Age (years)**					
18–29	78 (29%)	598	95	19.1	17.7
30–44	56 (21%)	577	84	24.8	18.9
45–55	133 (50%)	515	95	22.3	18.8
**Household income ($A)**					
< 80,000	101 (38%)	467	93	22.1	17.7
> = 80,000	103 (39%)	755	93	24.3	18.9
Prefer not to say	63 (24%)				

^a^For most people, a BMI of 18.5 to 24.9 is within the healthy weight category

## Results

### Descriptive data

Table 3 shows the characteristics of the 267 participants. The high proportion of female and older participants is not an issue for equity analyses, because changes in outcome measures are averaged for each population group. Only 204 participants reported their income, so analyses involving income are limited to this subsample. The majority of participants reported at least 150 min of moderate to vigorous activity, which the Department of Health [49] considers to be sufficient weekly physical activity for adults aged 18–64. Of the 267 participants, 229 reported at least one commuting trip and 259 reported at least one non-commuting trip. Between them, they reported 4,936 trips.

### Predictive transport mode choice model results

The final transport mode choice models for commuting and non-commuting are presented in Table 4. The models are a significant improvement over constants only ones (*p* < 0.01) and fit the data well (pseudo-R^2^ ≥ 0.56).

**Table 4:** Mixed logit models of transport mode choice

	Commuting (1,788 trips)			Non-commuting (3,148 trips)		
	β	95% confidence interval	t-statistic	β	95% confidence interval	t-statistic
*Alternative-specific constants*						
Bicycle	-4.92	-5.62 to -4.21	**-13.66**	-2.77	-2.99 to -2.55	**-24.86**
Public transport	-4.70	-5.55 to -3.85	**-10.83**	-5.59	-6.02 to -5.16	**-25.25**
Car	-2.79	-3.41 to -2.16	**-8.76**	-2.61	-2.81 to -2.41	**-25.96**
*Random parameters (specific alternative)*^*a*^						
Cycleway distance (Bicycle)	-1.89	-2.16 to -1.6	**-13.15**	-2.07	-2.35 to -1.78	**-14.24**
Non-cycleway distance (Bicycle)	-2.65	-3 to -2.3	**-15.05**	-3.35	-3.84 to -2.86	**-13.37**
CBD (Bicycle)	-1.52	-2.06 to -0.98	**-5.52**	-2.31	-3.18 to -1.44	**-5.19**
Time (Walk)	-0.41	-0.45 to -0.37	**-22.88**	-0.37	-0.39 to -0.36	**-49.39**
Time (Public transport)	-0.44	-0.46 to -0.41	**-40.26**	-0.32	-0.34 to -0.29	**-25.02**
Time (Car)	-1.17	-1.23 to -1.10	**-36.13**	-0.76	-0.79 to -0.73	**-47.47**
CBD (Car)	-5.66	-6.18 to -5.13	**-21.07**	-3.71	-4.21 to -3.2	**-14.39**
*Non-random parameters (specific alternative)*						
Non-cycleway distance x Low intensity (Bicycle)	-1.16	-1.38 to -0.92	**-9.68**	-0.99	-1.25 to -0.74	**-7.76**
Rain > 3 mm (Bicycle)				-0.79	-1.16 to -0.43	**-4.26**
*Model fit statistics*						
Log likelihood	-2478.7			-1928.8		
Chi-square	**2887.5 (*p* < 0.01)**			**4878.7 (*p* < 0.01)**		
Degrees of freedom	11			12		
Pseudo-R^2^	0.58			0.56		
Akaike information criterion	2091.9			3881.6		

^a^All random parameters have a triangular distribution with spread equal to half the mean

The two bicycle distance parameters have the expected negative sign. As expected, cycleway distance is preferred over non-cycleway distance, and the difference between them is statistically significant in both models (t-statistic ≥ 2.36). The marginal rates of substitution (β_Non-cycleway distance_ / β_Cycleway distance_) indicate that people will take a longer, less direct route to ride on cycleways instead of amongst traffic. On average, they will opt to ride for up to 1.4 km (commuting) or up to 1.6 km (not commuting) on cycleways, rather than ride for 1 km on facilities not protected from traffic.

Self-reported bicycle rider type has a significant influence on sensitivity to non-cycleway distance (i.e., aversion to cycling in traffic), with respondents identifying as ‘low intensity’ having a higher sensitivity.

The parameters for household income, education level, gender and age are not statistically significant; therefore, these variables are omitted in the final models.

### Physical activity forecasts

**Fig. 3:**
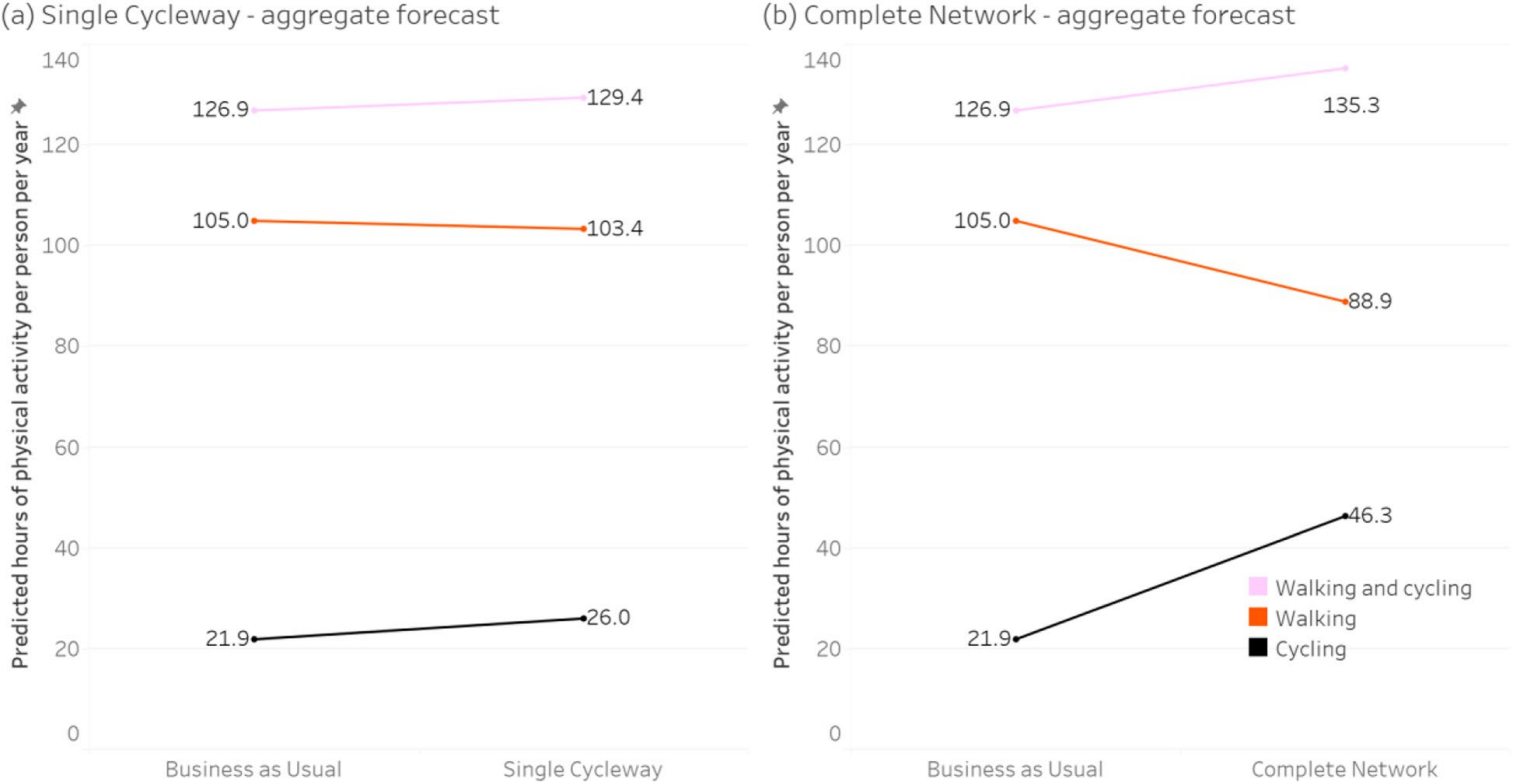
Forecast changes in physical activity – aggregated

**Fig. 4:**
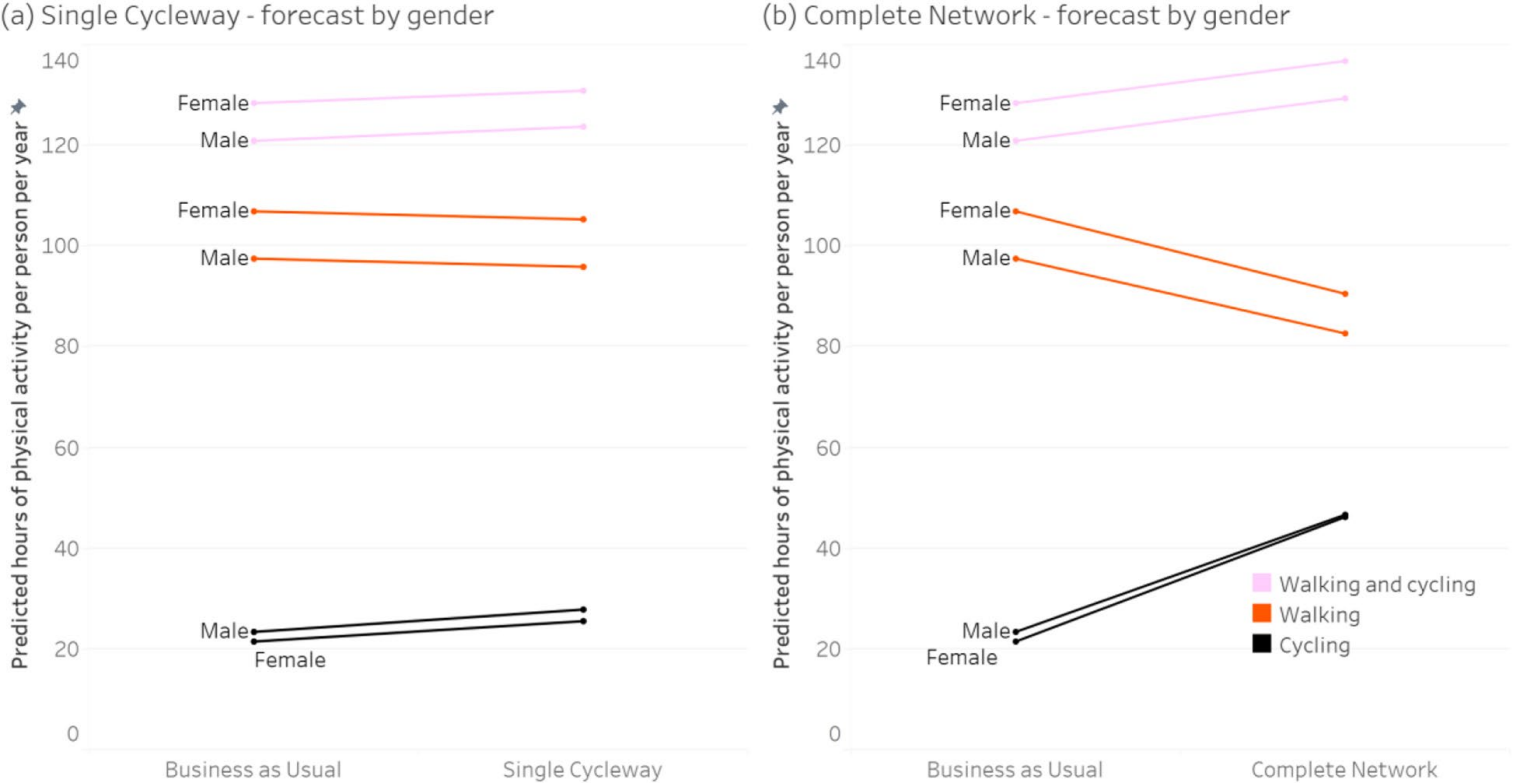
Forecast changes in physical activity – grouped by gender

**Fig. 5:**
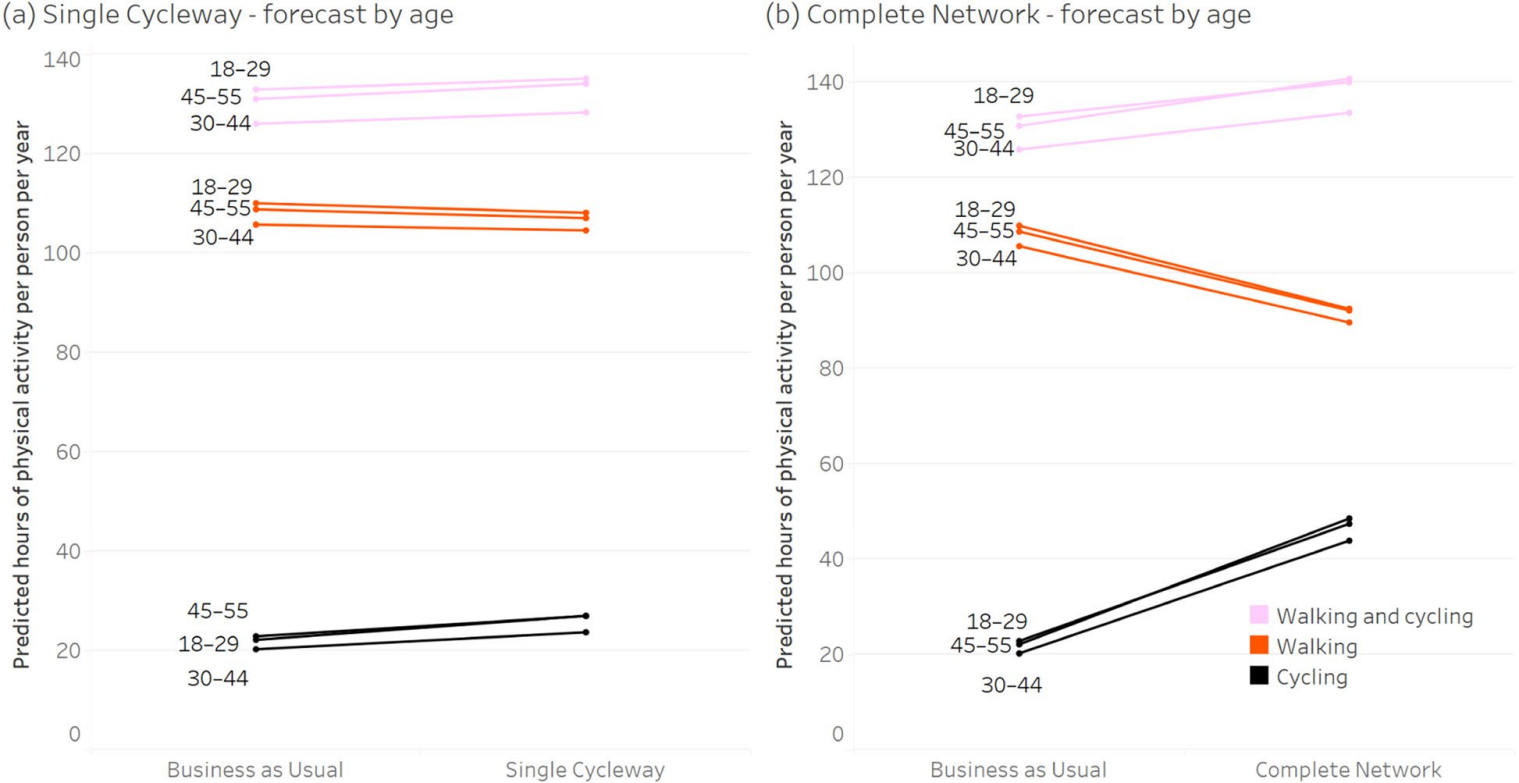
Forecast changes in physical activity – grouped by age

**Fig. 6:**
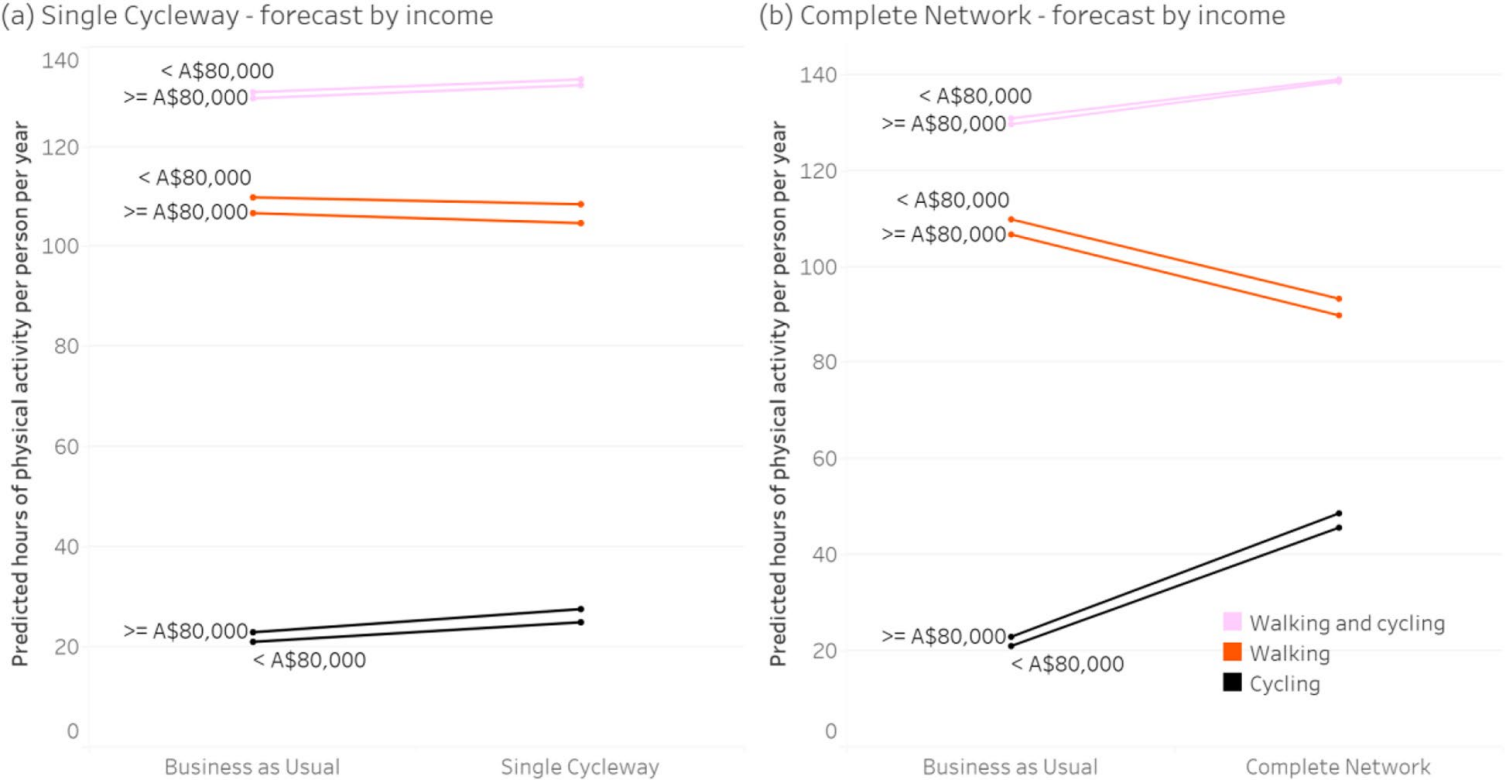
Forecast changes in physical activity – grouped by income

Figures 3, 4, 5 and 6 show the forecast hours of physical activity per person per year for the two intervention scenarios, relative to *Business as Usual*. In the *Single Cycleway* scenario, average cycling hours per person per year are forecast to increase by 18.9%. The forecast increase for males (19.0%) is almost identical to that for females (18.9%) and marginally greater for the high-income group (20.2% versus 18.6%) and the 45–55 age group (21.8% versus 17.8% for the 18–29 age group and 16.9% for the 30–44 age group).

In the *Complete Network* scenario, average cycling hours are forecast to more than double (111.7% increase). As with the *Single Cycleway* scenario, the forecast increase is greatest for the 45–55 age group (119.1%). However, in this scenario, the forecast increase is greater for females than males (115.1 versus 99.5%) and greater for the low-income group than the high-income group (117.2 versus 112.0%).

In the *Complete Network* scenario, the average cycling time for females (53.3 min/week) is brought almost to the same level as that for males (53.9 min/week). This finding could be partly explained by the transport mode choice model – which indicates people identifying as low-intensity bicycle riders have a greater aversion to riding amongst traffic – and the high correlation between respondents identifying as low intensity and female (*Χ*^*2*^ = 26.4, *p* < 0.001). Similarly, the greater physical activity gains for the 45–55 age group in the *Complete Network* scenario could be due to respondents in this group being more likely to identify as low-intensity bicycle riders (*Χ*^*2*^ = 10.5, *p* = 0.001) and having a greater aversion to riding amongst traffic.

In both intervention scenarios, increases in cycling time for all groups are partially offset by forecast decreases in walking time – which can be attributed to (a) some of the new cycling trips having previously been made by walking, and (b) those new cycling trips having a shorter travel time than the walking trips they replace, owing to the higher speed of bicycle (assuming destination choice is independent of transport mode choice).

However, in both intervention scenarios, there is still an increase in combined walking and cycling time: 2.0% in the *Single Cycleway* scenario and 6.6% in the *Complete Network* scenario, albeit with little difference between gender, age and income groups.

**Fig. 7:**
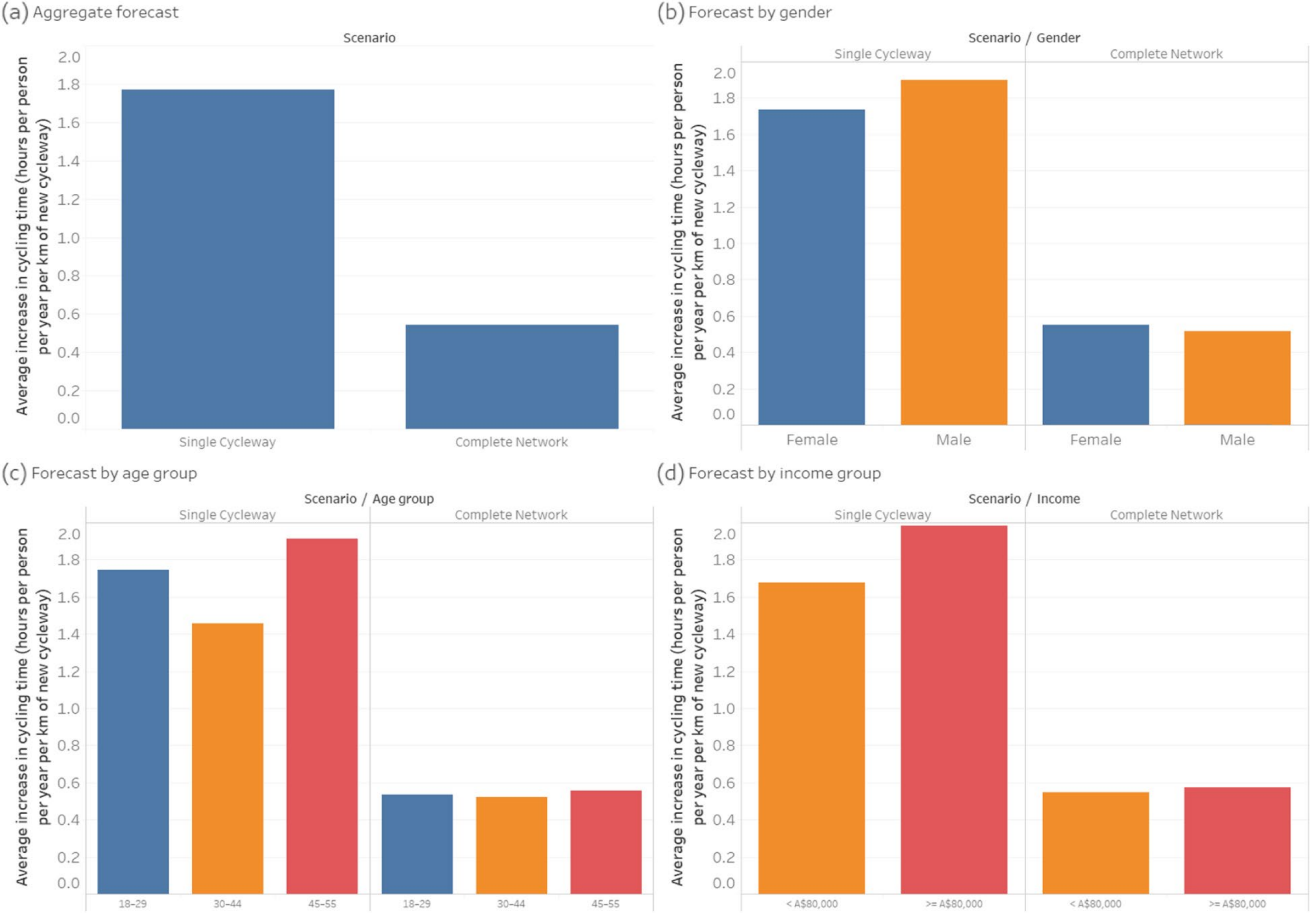
Forecast increase in cycling hours

Figure 7 shows the forecast increase in cycling physical activity per person per year for each kilometre of new cycleway built. In the Single *Cycleway Scenario*, each new kilometre of cycleway is forecast to result in greater increases in cycling physical activity among males than among females, among the 18–29 and 45–55 age groups, and among the high-income group. In the *Complete Network* scenario, forecast increases in cycling physical activity are roughly equal for all groups. While the *Complete Network* scenario is forecast to have a greater overall cycling physical activity benefit than the *Single Cycleway* scenario, the benefit per new cycleway kilometre is less, indicating diminishing returns as the network grows.

### Accessibility forecasts

**Fig. 8:**
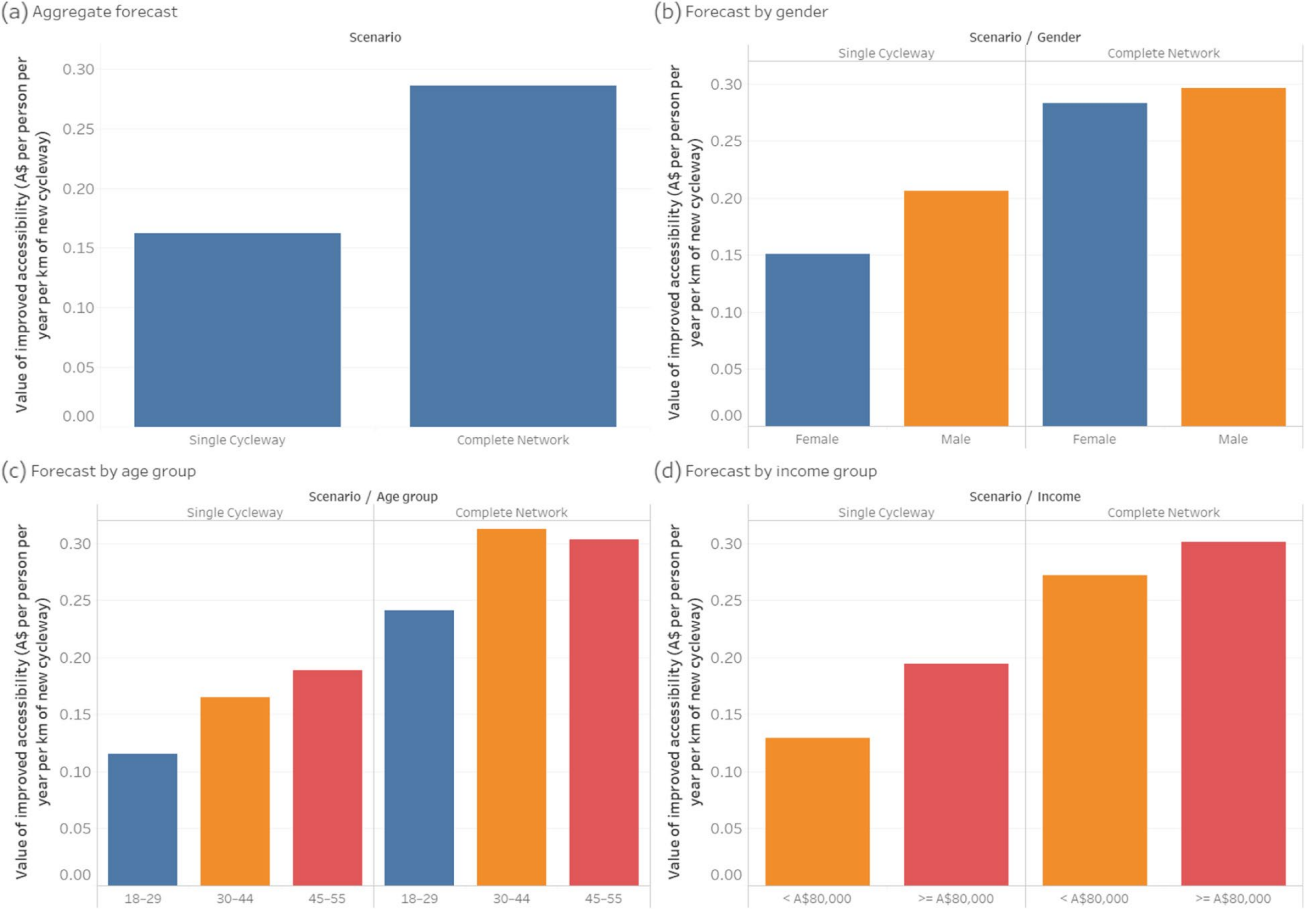
Forecast value of improved accessibility

Figure 8 shows the forecast value of accessibility improvements per person per year for each kilometre of new cycleway built. Overall, the *Complete Network* scenario has a 76% greater accessibility benefit per cycleway kilometre than the *Single Cycleway* scenario.

With the *Single Cycleway*, the accessibility benefit per cycleway kilometre is 37% greater for males than females, and 50% greater for the high-income group than the low-income group. These differences reduce to 5 and 11% respectively in the *Complete Network* scenario.

## Discussion

This study explored the potential impacts of new bicycle infrastructure on physical activity and accessibility across gender, age and income groups – for both a small-scale intervention (*Single Cycleway*) and a large-scale one (*Complete Network*).

The results suggest that: (a) the overall physical activity and accessibility benefits of new cycleways increase when they are joined into a fully-connected network that allows end-to-end, traffic-free cycling between multiple origins and destinations; (b) the accessibility benefits are amplified (due to network effects), but with a diminishing return in the physical activity benefits; and (c) the physical activity and accessibility benefits of new cycleways are much more equally distributed across population groups when they are joined into a complete network.

The forecasts are consistent with cycling participation data from other high-income countries, which show that, in cities with sparse/disconnected cycling infrastructure, transport cycling is predominantly an option for young and middle-aged adult males. Conversely, in cities with connected, low-stress bicycle networks, people of all genders and ages cycle for everyday transport [50–53].

The predictive transport mode choice model developed for this study reflects previous studies indicating that people prefer cycling on protected cycleways over cycling in traffic, and will take a less direct/more time-consuming route to do so [54–56]. That people willingly choose a longer route for greater journey utility raises questions about the implied objectives of speed increases and ‘travel time savings’ in much traffic engineering and transport planning practice and research [57].

Using our model to forecast the physical activity benefits of two bicycle infrastructure intervention scenarios, we estimate the *Single Cycleway* would increase average weekly cycling time per person from 25 to 30 min, while the *Complete Network* would more than double it, to 53 min. In both scenarios, some walking trips would be replaced by cycling trips (of similar distance and therefore reduced duration), resulting in a reduction in walking time. However, there is still a net increase in average weekly walking and cycling time, from 146 to 149 min in the *Single Cycleway* scenario, and to 156 min in the *Complete Network* scenario. In practice, a person switching from walking to cycling may opt for a more distant destination, because the higher speed of bicycle means they can access it in a similar time [58]. Accordingly, these forecast physical activity increases are likely to be conservative.

For reference, Australia’s Department of Health recommends a minimum of 150 min of moderate-intensity physical activity per week [59], which is in accordance with World Health Organization guidelines [60]. While (non-brisk) walking is not considered in the guidelines to be a moderate-intensity activity, cycling is. Thus, in the *Complete Network* scenario, the proportion of the recommended 150 min that could be achieved through transport cycling alone would increase from 17 to 35%.

Females and older adults in Australia are more likely to be inactive or only moderately active [61], which increases their risk of heart disease, type II diabetes and some cancers [62]. As such, the finding that, in the *Complete Network* scenario, greater physical activity benefit accrues to females and the 45–55 age group is encouraging from a health equity perspective. However, it should be noted that, in our sample, 95% of females were already sufficiently physically active to begin with.

The greater accessibility benefit forecast for the high-income group and the older age group can be partly explained by the greater number of trips reported by these groups (see Table 3), and we have assumed that the number of trips each person makes would be the same in all scenarios. In practice, new bicycle infrastructure may enable people in the other groups to make more trips.

The disaggregate transport demand forecasting model used for this study (in which forecasts are made for individuals with linked sociodemographic characteristics) enables greater insight into health equity outcomes than would be possible using the type of aggregate demand model typically used by transport authorities [46]. Our model also uses a finer spatial resolution than typical aggregate demand models (in which trip origins and destinations are approximated to zone centroids), allowing improved modelling of short-distance walking and cycling trips. However, like all predictive models, it involves many assumptions and several limitations. We have assumed that changes in a person’s cycling physical activity resulting from an infrastructure intervention may affect only their walking physical activity. However, it is also possible that a person spending more time cycling may replace other types of physical activity, e.g., working out at a gym. A systematic review of studies of the impact of built environment changes on physical activity and active transport [63] found largely positive effects for cycling physical activity, but was inconclusive in relation to overall physical activity.

We have also assumed that only transport mode choice and bicycle route choice would be affected by a bicycle infrastructure intervention. However, it is also likely that home location, work location, number of trips, departure time and destination choices would also be affected. For example, a person switching from driving to cycling for grocery shopping may opt for more frequent trips to a closer supermarket without car parking. Likewise, a person financially constrained from using public transport may make more trips and visit more distant destinations, given the option to get around by bicycle.

Achieving recruitment quotas for some population groups proved challenging, resulting in a convenience sample not representative of the population on certain demographics. While changes in outcome measures were averaged for each population group, the under-representation of some population groups may have biased the calculations. Furthermore, more people with a predisposition to healthy and active living may have self-selected for a survey about transport and health.

The forecast benefits are likely to be conservative because they do not include those accruing to people living outside the City of Sydney LGA, nor people aged less than 18 or more than 55 years. Nor do they include potential benefits associated with increased recreational cycling or improved opportunities to access or egress public transport (i.e., to get from a trip origin, e.g., home, to a public transport stop, or to get from a public transport stop to a destination, e.g., work).

Data were collected before the introduction of dockless bicycle share and widespread e-bike adoption in Sydney. While riding an e-bike is still a form of physical activity, it is generally lower intensity than riding a conventional bicycle; however, e-bike use is associated with more overall minutes of physical activity because users cycle more frequently and further than they would otherwise [64]. Future transport demand models used to predict impacts of bicycle infrastructure could include e-bike as a distinct transport mode alternative, or as an attribute in the bicycle alternative.

Despite the limitations and conservative estimates of this study, we have demonstrated how the distributions of physical activity and accessibility benefits of bicycle infrastructure can be assessed. While the findings are specific to inner-city Sydney, where most everyday destinations are within cycling distance, the method could be used anywhere that disaggregate transport demand data linked with personal characteristics are available.

The study indicates that bicycle infrastructure projects are likely to improve physical activity and accessibility for some population groups more than others, but that the benefits may be more evenly distributed with a fully connected, low-stress bicycle network. Thus, it could be argued that failure to provide a connected, low-stress bicycle network is an example of structural discrimination [65], as doing so limits the physical activity and independent access opportunities of females and other population groups most averse to cycling in traffic.

As such, we suggest that planning and assessment of major bicycle projects in future should consider the distributions of key benefits (and costs), especially (a) how much physical activity benefit accrues to population groups with higher incidence of inadequate physical activity (these being females and older adults in Australia); and (b) to what extent they could narrow existing disparities between population groups in opportunities to access economic/social opportunities and services.

## Conclusions

In a traffic-dominated city such as Sydney, certain population groups, notably females, have less opportunity to access everyday destinations by bicycle – and, therefore, incorporate moderate/high-intensity physical activity into their daily schedules – because of their greater aversion to riding in traffic. This inequity can be addressed with connected bicycle networks that provide more opportunities for people of all genders, ages and incomes to cycle to multiple destinations in a traffic-free environment.

## Data Availability

The datasets used and/or analysed during the current study are available from the corresponding author on reasonable request.

## Abbreviations

BMI: Body mass index
CBD: Central business district
LGA: Local government area
NSW: New South Wales
